# Turning over New Leaves

**Published:** 1889-04-06

**Authors:** 


### Turning Over New Leaves.

(Books for Review should be sent to The Editor, The Lodge, Porchester Square, W.)

### Guide to Stretcher and Bearer Company Drill.*

This guide may be used to supply the place of the "Manual for the Medical Staff Corps," now out of print, so far as it deals with special drill for stretchers, wagons, cacolets, litters, and hand seats. Part 1 treats of "duties, establishment, and administration." A battle is supposed to be taking place, and a wounded soldier is traced from the field to his ultimate resting-place at the base hospital. The man, who falls wounded at the fighting line, is attended to by the regimental surgeon, and then carried by the regimental bearers, or men of the bearer company of the Medical Staff Corps, to the collecting station in the rear. From the collecting station the wounded man is taken in an ambulance to the dressing station, which should be half a mile to the rear. Here the patient is again examined and treated by the medical officers in charge. From the dressing station the wounded man is taken, in the second line of ambulances, to the field hospital, a longer distance in the rear. From the field hospital the patient is eventually conveyed to the stationary hospital. Through each stage of progress a memo. accompanies concerning the patient's wound, treatment, name, etc. Staff-Sergeant Waterson does not forget to describe in detail all the appliances which each person or party engaged in the work as above should have in possession. Plates showing the position of the different halting places and hospitals are given. And the second part of the book is an elaborate detail of bearer company drill. Now all this looks facile on paper, but in actual practice in time of war it is not quite so easy. The whole system may be rehearsed on a flat parade ground, with sham patients and no danger; but when the din, and smoke, and excitement, and probably loss of assistance from assistants being wounded, occurs, the circumstances and position are altogether altered. In our opinion the tendency of recent years has been to render the system of attending to the wounded in battle too complex. The bearer company drill given by Staff-Sergeant Waterson runs through a hundred pages, and, although certainly the pages are not very large, the drill is a study of itself. Then the author also advises our acquaintance with Surgeon-Major Evatt's "Plan of the Medical Arrangements for an Army Corps in the Field," which acquaintance, we can say from our own knowledge, is not to be obtained by a glance. In addition to this, nine other books are named, the study of which is recommended. We are of opinion that the treatment of the wounded during and after battle should be propounded by any single or private individual, and that there should only be one bearer company drill, to be rendered as simple as possible. Mr. Waterson says his "drill is quite a new one"; and if so, it must evidence greater excellencies than we perceive to supplant that already taught in the Army Medical Staff Corps. Moreover, we do not quite understand why Mr. Waterson should publish a book of the kind, for the duties, establishment, and administration of the Army Medical Department are all detailed in the "Regulations of H.M.'s Army for 1885," from which, indeed, this section of Mr. Waterson's work has been taken. The book may be useful to amateur soldiers not possessing the Army Regulations, and in this manner may fill a void. It may also be useful in directing attention to the important part the medical staff plays during and after an engagement. When the soldier's work is done the doctor's work begins; but, as a rule, the world hears little of it. The army surgeon is usually only rewarded when he leaves his own duties and takes a sword in hand. But the art of killing has always been considered more honourable than the art of curing, even although statistics prove the latter to be the most dangerous of the two. It is a satire on our pseudo-civilisation that we are not in this respect advanced beyond the savages of antiquity.

* Guide to Stretcher and Bearer Company Drill. By Staff-Sergeant W. K. Waterson, First Division Vol. Med. Staff Corps.

